# Severe Acute Kidney Injury Secondary to Rhabdomyolysis in Diabetic Ketoacidosis (DKA): A Case Report

**DOI:** 10.7759/cureus.30619

**Published:** 2022-10-23

**Authors:** Zahidah A AL Aldahan, Saqib Ali, Fatimah A Al Aldahan, Doaa M AL Aldahan, Bashier A Al Aldahan

**Affiliations:** 1 Internal Medicine, King Salman Hospital, Riyadh, SAU; 2 Immunology and infection, University of Alberta, Alberta, CAN; 3 Clinical Research, King Fahad Medical City, Riyadh, SAU; 4 Emergency Department, Qatif Central Hospital, Qatif, SAU

**Keywords:** acute renal failure, hypophosphatemia, rhabdomyolysis, dka, diabetic emergencies

## Abstract

Delayed diagnosis and treatment of rhabdomyolysis in diabetic emergencies may lead to irreversible kidney damage and progress to chronic kidney disease. Therefore, early detection and correction of electrolyte disturbances, resulting from diabetes and risk factors for rhabdomyolysis, is essential to avoid complications and renal function improvement. In this case report, a patient with two weeks history of polyuria, polydipsia, and nocturia showed up at ED with epigastric abdominal pain, nausea, and non-bloody vomiting. The patient was in a diabetic ketoacidosis episode in which rhabdomyolysis developed and complicated into acute renal failure. Few case reports in the literature have mentioned the association of hypophosphatemia, severe acidosis, and high osmolarity as contributors to rhabdomyolysis leading to acute kidney injury (AKI) in patients with hyperglycemic emergency cases. To our knowledge, this is the first case reported in Saudi Arabia. Therefore, our unique case sheds some light on an overlooked complication in diabetic ketoacidosis (DKA), rhabdomyolysis, in which electrolyte abnormalities are the most probable trigger.

## Introduction

Rhabdomyolysis (RM) is sarcolemma disintegration and release of its contents into blood [1]. These include electrolytes, myoglobin, and creatinine phosphokinase (CPK) [2]. This damage could baffle other physiological systems inducing kidney and electrolyte homeostasis [2]. Etiologically, diabetic ketoacidosis (DKA) is a nontraumatic genesis for RM [3], however, a disregarded one as the severity of RM in DKA goes from asymptomatic to being mortal [4]. DKA mediation in muscle damage is not clearly determined [4]. Potential mechanisms are energy depletion, Na+/K+ pump disruption, developing hypophosphatemia, and hypokalemia [3,4]. RM is an infrequently reported complication of DKA [4]; therefore, highlighting such cases is significant since diabetes has a high prevalence. Here is a case of DKA in which rhabdomyolysis developed, and complicated to acute renal failure (ARF).

## Case presentation

**Figure 1:**
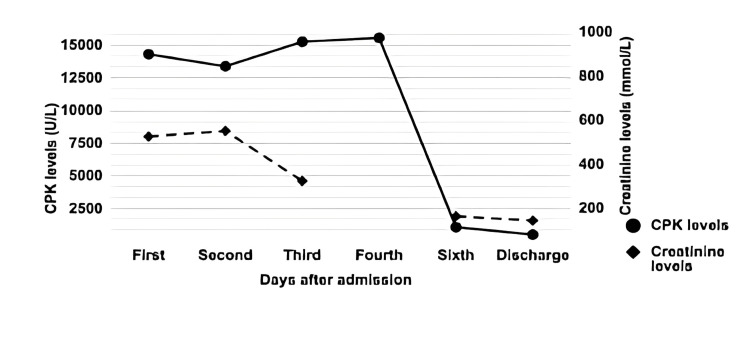
Elevation of the CPK levels (normal range 26-308 U/L) and creatinine levels (normal range 35-150 mmol/L) from day one in ICU to the day of discharge. Levels of CPK 5x higher than the upper limit of the normal range are strong indicators for RM; usually, its levels peaks at day 3 to day 5 and return to baseline levels the following 6-10 days. Both creatinine and CPK are markers for ARF when their levels increase. CPK creatine phosphokinase, RM rhabdomyolysis, ARF acute renal failure

A 31-year-old male presented to the emergency room with a two-week history of polyuria, nocturia, and polydipsia. The patient was complaining of mild epigastric abdominal pain associated with nausea and vomiting (10x in one day). The patient denied previous history of drugs, alcohol, or strenuous work. On admission, the patient was hyperglycemic, had severe metabolic acidosis, and severe respiratory distress. Venous blood gas (VBG) and laboratory evaluation revealed a severe episode of DKA (anion gap 18.9 mEq/L, pH < 7.3, serum bicarbonate 5.9 mEq/L, serum ketones 5.2 mmol.L) (Table 1). DKA protocol was followed to manage the case. However, the patient's condition rapidly deteriorated, and his vital signs became unstable (low Glasgow Coma Scale (GCS) and low blood oxygen); subsequently, he was intubated (BP improved to 106/65 mmHg, pulse to 112 breaths/min, RR to 20 breaths/min, and O₂ sat. to 96%) and transferred to the Intensive Care Unit (ICU) for further monitoring and management where a fluid replacement, insulin therapy, and intravenous sodium bicarbonate continued according to the DKA protocol. Laboratory examination showed leukocytosis and thrombocytopenia, though no evidence of infections was identified. Physical examination was unremarkable other than acanthosis nigricans evident over the back of the neck. Other examinations such as pan culture (blood, urine, methicillin-resistant Staphylococcus aureus (MRSA) nasal, rectal carbapenem-resistant Enterobacteriaceae (CRE), and endotracheal tube cultures) and radiological images (chest X-ray, CT brain, abdominal ultrasound, electrocardiogram, and echocardiogram) were all normal. The electrolyte profile displayed hypophosphatemia, elevated levels of CPK, and elevated levels of creatinine. After 19 hours of admission, the patient was out of ketosis, however, continued to have acidosis. Notably, the prolonged acidosis and the severe dehydration the patient had were suggestive of the acute renal failure he developed (elevated levels in blood urea nitrogen (BUN) 18.5 mmol/L, baseline 2.6-6.2 mmol/L; glomerular cast ++; protein and glucose in the urine) (Table 2, Figure 1). Intravenous phosphate replacement was administered, conventional renal replacement therapy was initiated, and the renal impairment was successfully revised. The patient’s condition improved after a six-day stay in the ICU (Table 2, 3). The patient was discharged on a subcutaneous insulin basal/bolus regimen and followed up with a diabetic care clinic.

**Table 1:** Laboratory findings and Investigations WBC white blood cell, CBC complete blood count, VBG Venous blood gas, HGB hemoglobin, PLT Platelet, NUT neutrophils, LYMP lymphocytes, PH potential of hydrogen, pCO2 partial pressure of carbon dioxide, pO2 partial pressure of oxygen, HCO3 Bicarbonate, ALT alanine aminotransferase, AST aspartate transaminase, NA sodium, K Potassium Cl chlorine, Mg+ Magnesium, Ca Calcium, LDL low-density lipoprotein, HDL high-density lipoprotein, PT prothrombin time, INR international normalized ratio, PTT partial thromboplastin time, RBC red blood cell, HPF Hematopoietic-promoting factor.

	Lab test	Patient result at admission	After 19 hours	Patient result at days after
CBC results	WBC (4-11)	28.4	15.5	11.6
	HGB (14-18 mg/dl)	15.1	15.1	14.4
	PLT (150-450)	115	123	175
	NUT (40-70%)	82.1%	12.1%	-
	LYMP (20-45%)	8.7%	0.7%	-
VBG results	PH	6.9	7.05	7.35
	pCO2 (mmHg)	19.3	20.6	22.5
	pO2 (mmHg)	62.8	64	136
	Potassium (mmol/l)	4.3	4.8	2.3
	Sodium (mmol/l)	119	131	158
	HCO3	5.9	11	25
	Blood ketones (mmol/l)	5.2	0.3	0.1
	Anion gap (mEq/L)	18.9	19.9	Normal
Biochemistry	Amylase (25-115 U/L)	1636	-	188 (then normalized in the following days)
	ALT(30-65)	41	-	-
	AST (15-37)	181	-	-
	lipase	N/A	-	-
	Troponin	Negative	-	-
Electrolyte profile	Na (132-145 mmol/l)	132	-	-
	K(3.2-5.5mmol/l)	4.8	-	-
	Cl(98-110 mmol/l)	105	-	-
	Mg+(0.74-0.99mmol/l)	1.01	-	-
	Ca (2.1-2.6mmol/l)	1.9	-	-
	Osmolality (275-305mmol/l)	289.1	-	-
Lipid profile	Triglyceride ( mmol/L)	2.1	-	-
	LDL (mmol/L)	3	-	-
	HDL (mmol/L)	1.55	-	-
	Cholesterol (mmol/L)	5.52	-	-
Coagulation profile	PT (10-15sec.)	12	-	-
	INR (0.9-1.2)	1	-	-
	PTT (28-40 sec)	29.2	-	-
Urinalysis	Appearance and color	Light brown		
	Chemical	PH		acidic
		Protein		+
		Glucose		+++
		Blood		+++
		Keton		+++
	Microscope & Urine Sediment	RBC		1-2/HPF
		Epithelial cell		+/HPF
		Glomerular cast		++
		Bacteria		+

**Table 2:** Laboratory findings and Investigations from day one at the ICU to discharge CPK Creatine phosphokinase, BUN blood urea nitrogen, PO+4 phosphate

Day	1	2	3	4	6	Discharge
CPK (26-308U/L)	14330	13398	15277	15572	1087	508
BUN (2.6-6.2mmol/L)	18.5	20.2	11.5	11.5	9.7	5.9
Creatinine (35-150 mmol/L)	526	552	324	-	164	144
PO+4 (0.81-1.5 mmol/L)	0.2	0.36	0.48	-	-	-
Serum Lactate	0.3	0.52	0.45	0.98	-	-

**Table 3:** Input & output CRRT Continuous renal replacement therapy

Day	1	2	3
Input	9L	7L	4.5L
Output	300L	700ml	Anuric (CRRT)

## Discussion

Rhabdomyolysis is a rare condition, thus there is not enough data regarding its prevalence as a complication in DKA [4]. It may be mainly due to the ranges in symptoms from being asymptomatic to life-threatening, to the unclear association between the two disorders, or to the sporadic reported cases found in the literature [4]. In addition, a potentially lethal complication like acute renal failure is usually assumed to be due to diabetes or dehydration, but not RM [1]. Consequently, there is a likelihood for RM diagnosis in DKA to be missed [4]. In this case, the clinical characteristics of rhabdomyolysis suggest its classification as nontraumatic, electrolytic, and metabolic in etiology. This diagnosis was based on indicative criteria such as CPK levels, which were elevated about 46x the normal levels (26-308 U/L). Consistently, significant muscle damage is deemed when CPK levels are at 5,000 U/L [3,5,6]. Other signs found are tea-colored acidic urine and red blood cells and protein in the urine. The underlying mechanism of RM in this case is unclear, however, we hypothesize it to be the succession of multiple events. Insulin deficiency promotes hyperglycemia and intracellular energy depletion which, in turn, activates the insulin-antagonistic hormones such as glucagon. The ratio of these hormones to insulin is higher, leading gluconeogenesis and glycogenolysis to be stimulated in the liver. Likewise, lipolysis is stimulated in adipose tissue, releasing free fatty acids into the circulatory system and driving excess beta-oxidation in the liver. Consequently, ketone bodies are generated in surplus amounts, and during hyperglycemic crises, their clearance is greatly reduced [7]. Subsequently, ketonemia and hyperglycemia promote osmotic diuresis, which baffles electrolyte homeostasis and induces dehydration. Additionally, peripheral tissues’ uptake of potassium dramatically decreased as potassium was lost via osmotic diuresis. Serum potassium is assumed to decrease even further after insulin initiation therapy because potassium influxes to cells, resulting in hypokalemia [8]. Interestingly, hypokalemia in this patient was not evident from the electrolyte profile (4.8 mmol/L), as it fell within the normal range (3.2-5.5mmol/L). Nonetheless, it is documented that the potassium levels generally are low in DKA even in normal or hyperkalemia laboratory work results [2,7]. Plasma phosphate is also presumed to decline significantly after insulin therapy administration (90% incidence) [8,9]. Potential factors for hypophosphatemia are prolonged acidosis, extracellular hyperosmolarity, phosphate deficient reabsorption and excretion, and intracellular shift of phosphate [10]. Mostly, DKA and rhabdomyolysis co-occurrence facilitated ARF incidence. ARF is frequently reported as a complication of RM (13%-50% in RM cases) [5]; moreover, the incidence of ARF in DKA is not only common but also an expected short-term outcome explicit as chronic kidney damage [11]. When sarcolemma disintegrates and releases its contents, fluids are retained within the damaged muscles leading to a decrease in intravascular volume [5]. Hypovolemia is presumed in our patient, given that he was in an episode of hyperglycemic crisis and dehydration. The circumstances of extracellular fluid depletion, collectively, stimulate the antidiuretic hormone to retain water by promoting renal vasoconstriction [5]. Unfavorably with the presence of RM, the homeostasis activation to maintain extracellular fluids worsened the case as the released myoglobin increased in concentration leading to its precipitation along renal tubules [5]. Furthermore, kidney filtration is impaired by the released electrolytes as they form complexes and deposit in renal tubules [5,6]. All this manifested as glomerular casts in the urinalysis results of our patient.

## Conclusions

Hypophosphatemia and hypokalemia are the most probable factors causing RM after electrolyte muddling episodes that resulted from DKA and insulin correction. An appropriate and timely DKA treatment could prevent fatal outcomes. Hence, revisiting the perspective of how common rhabdomyolysis incidence in DKA cases would improve the understanding of rhabdomyolysis pathophysiology, prevalence, and indications in diabetic patients. 
